# Entropy Production Rates of the Multi-Dimensional Fractional Diffusion Processes

**DOI:** 10.3390/e21100973

**Published:** 2019-10-05

**Authors:** Yuri Luchko

**Affiliations:** Department of Mathematics, Physics, and Chemistry, Beuth Technical University of Applied Sciences Berlin, Luxemburger Str. 10, 13353 Berlin, Germany; luchko@beuth-hochschule.de

**Keywords:** time-space-fractional diffusion equation, fundamental solution, Mellin-Barnes integral, Shannon entropy, entropy production rate

## Abstract

Our starting point is the *n*-dimensional time-space-fractional partial differential equation (PDE) with the Caputo time-fractional derivative of order β,0<β<2 and the fractional spatial derivative (fractional Laplacian) of order α,0<α≤2. For this equation, we first derive some integral representations of the fundamental solution and then discuss its important properties including scaling invariants and non-negativity. The time-space-fractional PDE governs a fractional diffusion process if and only if its fundamental solution is non-negative and can be interpreted as a spatial probability density function evolving in time. These conditions are satisfied for an arbitrary dimension n∈N if 0<β≤1,0<α≤2 and additionally for 1<β≤α≤2 in the one-dimensional case. In all these cases, we derive the explicit formulas for the Shannon entropy and for the entropy production rate of a fractional diffusion process governed by the corresponding time-space-fractional PDE. The entropy production rate depends on the orders β and α of the time and spatial derivatives and on the space dimension *n* and is given by the expression $\frac{\beta \;n}{\alpha \;t}$, *t* being the time variable. Even if it is an increasing function in β, one cannot speak about any entropy production paradoxes related to these processes (as stated in some publications) because the time-space-fractional PDE governs a fractional diffusion process in all dimensions only under the condition 0<β≤1, i.e., only the slow and the conventional diffusion can be described by this equation.

## 1. Introduction

One of the most prominent and broadly-recognized applications of Fractional Calculus (FC) is for description of the anomalous transport processes [1,2,3,4]. The basic FC model for the slow anomalous diffusion is the time-fractional partial differential equation (PDE) that interpolates between the time-independent Poisson equation and the diffusion equation. In Reference [5], the fundamental solution to the time-fractional diffusion equation with the Caputo fractional derivative of order β∈(0,1) and the spatial Laplace operator was shown to be non-negative and normalized. Thus it can be interpreted as a spatial probability density function evolving in time that provides a strong justification for the time-fractional diffusion equation with the time derivative of order β∈(0,1) to act as a model for a diffusion process. This process is anomalous and slow because the mean squared displacement of the diffusing particle behaves as $c_{\beta }\;t^{\beta },\;\beta \in \left(0,1\right)$ in contrast to the linear dependence of the mean squared displacement on time in the case of the conventional diffusion.

In analogy to the case of the slow anomalous diffusion, some FC models for the supper-diffusion (fast diffusion) processes were introduced in form of the time-, space, or time-space-fractional PDEs. In particular, the time-fractional diffusion-wave PDE with the Caputo fractional derivative of order β∈(1,2] and the spatial Laplace operator, which interpolates between the diffusion equation and the wave equation, was often discussed in the literature in connection with the supper-diffusion. However, it turned out that the fundamental solution to the *n*-dimensional time-space-fractional PDE with the Caputo time-fractional derivative of the order β,0<β<2 and the fractional spatial derivative (fractional Laplacian) of the order α,0<α≤2 fails to be non-negative for n≥2 for all 1<β<2,0<α≤2 [6]. This means that for n≥2 this equation with the time-fractional derivative of order β greater than one cannot serve as a model for any diffusion process but rather as a model for the damped waves propagation [7,8].

However, the situation in the one-dimensional case (n=1) is very different from the one in the multi-dimensional case (n≥2). As shown in Reference [9], the fundamental solution to the one-dimensional time-space-fractional PDE with the time-fractional Caputo derivative of order β∈(0,2] and the fractional spatial Riesz–Feller derivative of order α∈(0,2] and skewness θ (|θ|≤min{α,2−α}) is non-negative both for 0<β≤1,0<α≤2 and for 1<β≤α≤2, i.e., this equation can describe some diffusion processes also for β greater than one. The entropy and the entropy production rates of the processes governed by the one-dimensional time-fractional diffusion equation with the Caputo time-fractional derivative of order β∈[1,2] and the second spatial derivative (Laplace operator in the one-dimensional case) were discussed in [10,11]. According to References [10,11], the entropy production rate of the time-fractional diffusion equation increases with increasing of β from one (diffusion) to two (wave propagation) that results in the so called entropy production paradox. Many efforts were put into "resolving" of this paradox (see e.g., [12] and references therein). However, as mentioned above, this paradox does not appear in the dimensions two and three, i.e., in the cases that are important for applications. The paradox in the one-dimensional case is rather a mathematical caprice than a physical phenomenon and thus it has only very restricted—if any—relevance for applications.

In the literature, the entropy and the entropy production rates of the processes governed by some other particular cases of the time-space-fractional PDE with the Caputo time-fractional derivative of order β,0<β<2 and the fractional spatial derivative (fractional Laplacian) of order α,0<α≤2 have been considered. In References [13,14], the case of the one-dimensional space-fractional PDE with the first-order time derivative and fractional spatial derivative of order α,α∈(0,2] was analyzed in detail. In Reference [15], a closed form formula for the Shannon entropy of the fundamental solution to the one-dimensional neutral-fractional PDE with the fractional derivatives of the same order α,1≤α≤2 both in space and in time, was derived. The *n*-dimensional case of this equation was analyzed in Reference [7]. In Reference [16], it was shown that the entropy production rate of the fundamental solution to the one-dimensional α-fractional diffusion equation is exactly the same as in the case of the conventional diffusion equation. The α-fractional diffusion equation is a PDE with the Caputo time-fractional derivative of order α,0<α≤1 and the fractional spatial derivative of order 2α. Thus the quotient of the derivatives orders is one half, i.e., exactly the same as for the conventional diffusion equation. The case of the two-dimensional α-fractional diffusion equation was considered in Reference [17]. In the *n*-dimensional case, the entropy of the processes governed by the α-fractional diffusion equation was discussed in Reference [18].

The rest of the paper is organized as follows. In Section 2, we first remind the readers of derivation of the fundamental solution to the *n*-dimensional time-space-fractional PDE with the Caputo time-fractional derivative of order β,0<β<2 and the fractional spatial derivative (fractional Laplacian) of order α,0<α≤2 and then mention its properties that are used in further discussions. Section 3 contains the main results and presents a derivation of the closed form formulas for the Shannon entropy and the entropy production rates of the fractional diffusion processes that are governed by those *n*-dimensional time-space-fractional PDEs that possess non-negative fundamental solutions (0<β≤1,0<α≤2 for n∈N or 1<β≤α≤2 for n=1). The last section is dedicated to the conclusions.

## 2. Fundamental Solution to the Time-Space-Fractional PDE

In this section, for the sake of the reader’s convenience we provide a sketch of derivation of the Mellin-Barnes integral representation of the fundamental solution to the time-space-fractional PDE with the Caputo time-fractional derivative and the fractional Laplacian (see Reference [19] for details). This representation is valid for the derivatives orders β∈(0,2),α∈(0,2] [20]. However, our derivation method works only in the case β∈(0,2),α∈(1,2] and thus we first suppose these conditions to hold true and consider the multi-dimensional time-space-fractional PDE in the following form:

(1) $$D_{t}^{\beta }u\left(x,t\right)+{\left(-\Delta \right)}^{\frac{\alpha }{2}}u\left(x,t\right)=0,\;x\in R^{n},\;t>0,\;0<\beta <2,\;1<\alpha \leq 2,$$

where $D_{t}^{\beta }$ is the Caputo time-fractional derivative of the order β and ${\left(-\Delta \right)}^{\frac{\alpha }{2}}$ is the fractional Laplacian. The Caputo time-fractional derivative of order β>0 is defined by the formula

(2) $$D_{t}^{\beta }u\left(x,t\right)=\left(I_{t}^{n-\beta }\frac{\partial^{n}u}{\partial t^{n}}\right)\left(t\right),\;n-1<\beta \leq n,\;n\in N\;,$$

where $I_{t}^{\gamma }$ is the Riemann–Liouville fractional integral given by

$$\left(I_{t}^{\gamma }u\right)\left(t\right)=\left\{\begin{array}{c} \frac{1}{\Gamma (\gamma )}\int_{0}^{t}{\left(t-\tau \right)}^{\gamma -1}u\left(x,\tau \right)\;d\tau \;\mathrm{for}\;\gamma >0,\\ u(x,t)\;\mathrm{for}\;\gamma =0. \end{array}\right.$$

For a sufficiently well-behaved function $f\;:\;R^{n}\to R$ and for 0<α<m,m∈N and $x\in R^{n}$, the fractional Laplacian can be represented as a hypersingular integral [21]:

(3) $${\left(-\Delta \right)}^{\frac{\alpha }{2}}f\left(x\right)=\frac{1}{d_{n,m}\left(\alpha \right)}\int_{R^{n}}\frac{\left(\Delta_{h}^{m}f\right)\left(x\right)}{{\left|h\right|}^{n+\alpha }}\;dh$$

with the suitably defined finite differences operator $\left(\Delta_{h}^{m}f\right)\left(x\right)$ and the normalization constant $d_{n,m}\left(\alpha \right)$. The operator $\left(\Delta_{h}^{m}f\right)\left(x\right)$ can be chosen either in the non-centered form

Δhmf(x)=∑k=0m(−1)kmkf(x−h)

or in the centered form:

Δhmf(x)=∑k=0m(−1)kmkf(x−(m/2−k)h).

The normalization constant $d_{n,m}\left(\alpha \right)$ is given by the formula

$$d_{n,m}\left(\alpha \right)=\frac{\pi^{1+n/2}A_{m}\left(\alpha \right)}{2^{\alpha }\Gamma \left(1+\alpha /2\right)\Gamma \left(\left(n+\alpha \right)/2\right)\sin\left(\pi \alpha /2\right)},$$

where

Am(α)=∑k=0m(−1)k−1mkkα

in the case of the non-centered difference operator and

Am(α)=2∑k=0[m/2](−1)k−1mk(m/2−k)α

in the case of the centered difference operator. The representation (3) of the fractional Laplacian does not depend on m,m∈N provided α<m and is valid with the centered differences operator for all α>0 and even *m* or with the non-centered differences operator for all α>0 except of the case α=1,3,5⋯,2[m/2]−1.

It is worth mentioning that the fractional Laplacian ${\left(-\Delta \right)}^{\frac{\alpha }{2}}$ can be also interpreted as a pseudo-differential operator with the symbol ${\left|\kappa \right|}^{\alpha }$ ([21,22]):

(4) $$\left(F\;{\left(-\Delta \right)}^{\frac{\alpha }{2}}f\right)\left(\kappa \right)={\left|\kappa \right|}^{\alpha }\left(F\;f\right)\left(\kappa \right)\;,$$

where (Ff)(κ) is the Fourier transform of a function *f* at the point $\kappa \in R^{n}$ defined by

(5) $$\left(F\;f\right)\left(\kappa \right)=\hat{f}\left(\kappa \right)=\int_{R^{n}}e^{i\kappa \cdot x}f\left(x\right)\;dx\;.$$

In what follows we consider an initial-value problem for the Equation (1) with the Dirichlet initial conditions. For 0<β≤1, we pose an initial condition in the form

(6) $$u\left(x,0\right)=\varphi \left(x\right)\;,\;x\in R^{n}.$$

If 1<β<2, the second initial condition is added:

(7) $$\frac{\partial u}{\partial t}\left(x,0\right)=0\;,\;x\in R^{n}.$$

Because the initial-value problem (1), (6) (or (1), (6)–(7), respectively) is a linear one, its solution can be represented in the form

$$u\left(x,t\right)=\int_{R^{n}}G_{\alpha ,\beta ,n}\left(x-\zeta ,t\right)\varphi \left(\zeta \right)\;d\zeta ,$$

where $G_{\alpha ,\beta ,n}$ is the fundamental solution, i.e., the solution to the problem (1), (6) with the initial condition

$$u\left(x,0\right)=\prod_{i=1}^{n}\delta \left(x_{i}\right)\;,\;x=\left(x_{1},x_{2},\ldots ,x_{n}\right)\in R^{n}$$

or to the problem (1), (6)–(7) with the initial conditions

$$u\left(x,0\right)=\prod_{i=1}^{n}\delta \left(x_{i}\right)\;,\;x=\left(x_{1},x_{2},\ldots ,x_{n}\right)\in R^{n}$$

and

$$\frac{\partial u}{\partial t}\left(x,0\right)=0\;,\;x\in R^{n},$$

for 0<β≤1 or 1<β<2, respectively, with δ being the Dirac delta function.

To derive a close form formula for the fundamental solution, we apply the Fourier transform (5) with respect to the spatial variable to the Equation (1) and to the initial conditions (6) with $\varphi \left(x\right)=\prod_{i=1}^{n}\delta \left(x_{i}\right)$ and (7) (in the case β>1) and get the fractional ordinary differential equation (ODE)

(8) $$D_{t}^{\beta }{\hat{G}}_{\alpha ,\beta ,n}\left(\kappa ,t\right)+{\left|\kappa \right|}^{\alpha }{\hat{G}}_{\alpha ,\beta ,n}\left(\kappa ,t\right)=0,$$

and the initial conditions

(9) $${\hat{G}}_{\alpha ,\beta ,n}\left(\kappa ,0\right)=1$$

in the case 0<β≤1 or the initial conditions

(10) $${\hat{G}}_{\alpha ,\beta ,n}\left(\kappa ,0\right)=1,\;\frac{\partial }{\partial t}{\hat{G}}_{\alpha ,\beta ,n}\left(\kappa ,0\right)=0$$

in the case 1<β<2.

In both cases, the unique solution to the initial-value problem for the fractional ODE (8) with the initial conditions (9) or (10), respectively, has the form [23]:

(11) $${\hat{G}}_{\alpha ,\beta ,n}\left(\kappa ,t\right)=E_{\beta }\left(-{\left|\kappa \right|}^{\alpha }t^{\beta }\right)\;,$$

where the Mittag-Leffler function $E_{\beta }\left(z\right)$ is defined as follows:

(12) $$E_{\beta }\left(z\right)=\sum_{n=0}^{\infty }\frac{z^{n}}{\Gamma (1+\beta \;n)}\;,\;\beta >0,\;z\in C.$$

Because of the asymptotic formula [24]

(13) $$E_{\beta }\left(-x\right)=-\sum_{k=1}^{m}\frac{{\left(-x\right)}^{-k}}{\Gamma (1-\beta k)}\;+{O(|x|}^{-1-m}),\;m\in N,\;x\to +\infty ,\;0<\beta <2,$$

the right-hand side of the Equation (11) is from $L_{1}\left(R^{n}\right)$ as soon as α>1. Thus for 1<α≤2, we can apply the inverse Fourier transform to the Equation (11) and get the representation

(14) $$G_{\alpha ,\beta ,n}\left(x,t\right)=\frac{1}{{\left(2\pi \right)}^{n}}\int_{R^{n}}e^{-i\kappa \cdot x}E_{\beta }\left(-{\left|\kappa \right|}^{\alpha }t^{\beta }\right)\;d\kappa \;,\;x\in R^{n}\;,t>0\;.$$

Because the function $E_{\beta }\left(-{\left|\kappa \right|}^{\alpha }t^{\beta }\right)$ is a radial function, we can rewrite the Equation (14) in the form [21]

(15) $$G_{\alpha ,\beta ,n}\left(x,t\right)=\frac{{\left|x\right|}^{1-\frac{n}{2}}}{{\left(2\pi \right)}^{\frac{n}{2}}}\;\int_{0}^{\infty }E_{\beta }\left(-\tau^{\alpha }t^{\beta }\right)\tau^{\frac{n}{2}}J_{\frac{n}{2}-1}\left(\tau |x|)\;d\tau \;,|x\right|\neq 0,$$

whenever the integral at the right-hand side of (15) converges absolutely or at least conditionally. In this formula, $J_{\nu }$ denotes the Bessel function with the index ν.

In the case |x|=0 (x=(0,…,0)), the formula (14) takes the form

$$G_{\alpha ,\beta ,n}\left(0,t\right)=\frac{1}{{\left(2\pi \right)}^{n}}\int_{R^{n}}E_{\beta }{\left(-|\kappa \right|}^{\alpha }t^{\beta })d\kappa$$

that can be rewritten as

(16) $$G_{\alpha ,\beta ,n}\left(0,t\right)=\frac{1}{{\left(2\pi \right)}^{n}}\frac{2\pi^{\frac{n}{2}}}{\Gamma (\frac{n}{2})}\int_{0}^{\infty }E_{\beta }\left(-\tau^{\alpha }t^{\beta }\right)\;\tau^{n-1}\;d\tau$$

due to the well-known formula for the multi-dimensional integrals of the radial functions [21]. The integral at the right-hand side of (16) is convergent under the condition 0<n<α. Thus the fundamental solution $G_{\alpha ,\beta ,n}$ is finite at the point |x|=0 only in the one-dimensional case (remember the condition 1<α≤2) and has an integrable singularity for other dimensions.

Let us now consider the case x≠0 and apply the variables substitution $u=\tau^{\alpha }t^{\beta }$ in the integral at the right-hand side of the integral representation (15) to get the expression

(17) $$G_{\alpha ,\beta ,n}\left(x,t\right)=t^{-\frac{\beta n}{\alpha }}L_{\alpha ,\beta ,n}\left(z\right),\;z=\frac{\left|x\right|}{2t^{\frac{\beta }{\alpha }}}$$

with the auxiliary function

(18) $$L_{\alpha ,\beta ,n}\left(z\right)=\frac{{\left(2z\right)}^{1-\frac{n}{2}}}{\alpha {\left(2\pi \right)}^{\frac{n}{2}}}\int_{0}^{\infty }u^{\frac{n}{2\alpha }+\frac{1}{\alpha }-1}E_{\beta }\left(-u\right)J_{\frac{n}{2}-1}\left(2z\;u^{\frac{1}{\alpha }}\right)\;du.$$

This representation will be used in the further discussions of the properties of $G_{\alpha ,\beta ,n}$.

Now we employ the technique of the Mellin integral transform to deduce a Mellin-Barnes representation of the fundamental solution $G_{\alpha ,\beta ,n}\left(x,t\right)$. Recall that the Mellin integral transform of a function *f* is defined by the formula

(19) $$f^{*}\left(s\right)=\left(Mf\left(\tau \right)\right)\left(s\right)=\int_{0}^{\infty }f\left(\tau \right)\tau^{s-1}\;d\tau \;,\;\gamma_{1}<ℜ\left(s\right)<\gamma_{2}$$

and the inverse Mellin integral transform has the form

(20) $$f\left(\tau \right)=\left(M^{-1}f^{*}\left(s\right)\right)\left(\tau \right)=\frac{1}{2\pi i}\int_{\gamma -i\infty }^{\gamma +i\infty }f^{*}\left(s\right)\tau^{-s}\;ds\;,\;\tau >0\;,\gamma_{1}<ℜ\left(s\right)=\gamma <\gamma_{2}\;.$$

If we denote by $\overset{M}{⟷}$ the juxtaposition of a function *f* with its Mellin transform $f^{*}$ then the convolution theorem for the Mellin integral transform reads as

(21) $$\int_{0}^{\infty }f_{1}\left(\tau \right)f_{2}\left(\frac{y}{\tau }\right)\;\frac{d\tau }{\tau }\;\overset{M}{⟷}\;f_{1}^{*}\left(s\right)f_{2}^{*}\left(s\right)\;.$$

As we can see, the integral at the right-hand side of the Equation (15) can be interpreted as the Mellin convolution of the functions

$$f_{1}\left(\tau \right)=E_{\beta }\left(-\tau^{\alpha }\;t^{\beta }\right)\;and\;f_{2}\left(\tau \right)=\frac{{\left|x\right|}^{-n}}{{\left(2\pi \right)}^{\frac{n}{2}}}\;\tau^{-\frac{n}{2}-1}\;J_{\frac{n}{2}-1}\left(\frac{1}{\tau }\right)$$

evaluated at the point $y=\frac{1}{\left|x\right|}$.

By using the Mellin integral transform of the Mittag-Leffler function [25]

$$E_{\beta }\left(-\tau \right)\overset{M}{⟷}\frac{\Gamma (s)\Gamma (1-s)}{\Gamma (1-\beta s)},\;0<ℜ\left(s\right)<1,\;0<\beta <2,$$

the Mellin integral transform of the Bessel function [25]

$$J_{\nu }\left(2\sqrt{\tau }\right)\overset{M}{⟷}\frac{\Gamma (\nu /2+s)}{\Gamma (\nu /2+1-s)},\;-ℜ\left(\nu /2\right)<ℜ\left(s\right)<3/4,$$

and some elementary properties of the Mellin integral transform [25,26], we arrive at the formulas:

$$\begin{array}{c} f_{1}^{*}\left(s\right)=\frac{t^{-\frac{\beta }{\alpha }\;s}}{\alpha }\frac{\Gamma \left(\frac{s}{\alpha }\right)\Gamma \left(1-\frac{s}{\alpha }\right)}{\Gamma (1-\frac{\beta }{\alpha }\;s)}\;,\;0<ℜ\left(s\right)<\alpha \;,\\ f_{2}^{*}\left(s\right)=\frac{{\left|x\right|}^{-n}}{{\left(2\pi \right)}^{\frac{n}{2}}}{\left(\frac{1}{2}\right)}^{-\frac{n}{2}+s}\frac{\Gamma \left(\frac{n}{2}-\frac{s}{2}\right)}{\Gamma \left(\frac{s}{2}\right)}\;,\;\frac{n}{2}-\frac{1}{2}<ℜ\left(s\right)<n\;. \end{array}$$

Then the Mellin convolution theorem (21) and the Equation (20) for the inverse Mellin transform provide us with the following Mellin-Barnes integral representation of the fundamental solution $G_{\alpha ,\beta ,n}$:

(22) $$G_{\alpha ,\beta ,n}\left(x,t\right)=\frac{1}{\alpha }\frac{{\left|x\right|}^{-n}}{\pi^{\frac{n}{2}}}\frac{1}{2\pi i}\int_{\gamma -i\infty }^{\gamma +i\infty }\frac{\Gamma \left(\frac{n}{2}-\frac{s}{2}\right)\Gamma \left(\frac{s}{\alpha }\right)\Gamma \left(1-\frac{s}{\alpha }\right)}{\Gamma \left(1-\frac{\beta }{\alpha }s\right)\Gamma \left(\frac{s}{2}\right)}{\left(\frac{2t^{\frac{\beta }{\alpha }}}{\left|x\right|}\right)}^{-s}ds\;,$$

where $\frac{n}{2}-\frac{1}{2}<\gamma <\min\left(\alpha ,n\right)$. The variables substitution s→n−s in the integral at the right-hand side of (22) leads to an equivalent representation

(23) $$G_{\alpha ,\beta ,n}\left(x,t\right)=\frac{1}{\alpha }\frac{t^{-\frac{\beta n}{\alpha }}}{{\left(4\pi \right)}^{\frac{n}{2}}}\frac{1}{2\pi i}\int_{\gamma -i\infty }^{\gamma +i\infty }\frac{\Gamma \left(\frac{s}{2}\right)\Gamma \left(\frac{n}{\alpha }-\frac{s}{\alpha }\right)\Gamma \left(1-\frac{n}{\alpha }+\frac{s}{\alpha }\right)}{\Gamma \left(1-\frac{\beta }{\alpha }n+\frac{\beta }{\alpha }s\right)\Gamma \left(\frac{n}{2}-\frac{s}{2}\right)}{\left(\frac{\left|x\right|}{2t^{\frac{\beta }{\alpha }}}\right)}^{-s}ds\;$$

with max(n−α,0)<γ<n.

Comparing the last formula with the expression (17), we get the following Mellin-Barnes representation of the auxiliary function $L_{\alpha ,\beta ,n}$:

(24) $$L_{\alpha ,\beta ,n}\left(z\right)=\frac{1}{\alpha \;{\left(4\pi \right)}^{\frac{n}{2}}}\frac{1}{2\pi i}\int_{\gamma -i\infty }^{\gamma +i\infty }\frac{\Gamma \left(\frac{s}{2}\right)\Gamma \left(\frac{n}{\alpha }-\frac{s}{\alpha }\right)\Gamma \left(1-\frac{n}{\alpha }+\frac{s}{\alpha }\right)}{\Gamma \left(1-\frac{\beta }{\alpha }n+\frac{\beta }{\alpha }s\right)\Gamma \left(\frac{n}{2}-\frac{s}{2}\right)}z^{-s}ds\;,$$

where the parameter γ satisfies the inequalities max(n−α,0)<γ<n. Thus we can determine the Mellin integral transform of the function $L_{\alpha ,\beta ,n}$:

(25) $$L_{\alpha ,\beta ,n}\left(\tau \right)\overset{M}{⟷}\frac{1}{\alpha \;{\left(4\pi \right)}^{\frac{n}{2}}}\frac{\Gamma \left(\frac{s}{2}\right)\Gamma \left(\frac{n}{\alpha }-\frac{s}{\alpha }\right)\Gamma \left(1-\frac{n}{\alpha }+\frac{s}{\alpha }\right)}{\Gamma \left(1-\frac{\beta }{\alpha }n+\frac{\beta }{\alpha }s\right)\Gamma \left(\frac{n}{2}-\frac{s}{2}\right)}$$

that is valid if max(n−α,0)<ℜ(s)<n.

It is worth mentioning that both the representation (17) and the Equation (25) will play a decisive role for derivation of the closed form formulas for the Shannon entropy and the entropy production rate of the fractional diffusion processes. In particular, the representation (17) shows that the fundamental solution $G_{\alpha ,\beta ,n}$ depends on the similarity variable $z=\frac{\left|x\right|}{2t^{\frac{\beta }{\alpha }}}$ that in fact determines the form of the entropy production rate of the fractional diffusion processes.

Another important point is the non-negativity property of the fundamental solution. The time-space-fractional PDE governs a fractional diffusion process if and only if its fundamental solution is non-negative and can be interpreted as a spatial probability density function (pdf) evolving in time. As recently shown in [6], these conditions are satisfied for an arbitrary dimension n∈N if 0<β≤1,0<α≤2 and additionally for 1<β≤α≤2 in the one-dimensional case. In the next section, we restrict ourselves only to these cases and derive the explicit formulas for the Shannon entropy and for the entropy production rate of the fractional diffusion processes governed by the corresponding time-space-fractional PDEs.

For further properties and numerous particular cases of the fundamental solution $G_{\alpha ,\beta ,n}$ we refer the interested readers to [19].

## 3. The Entropy Production Rates of the Fractional Diffusion Processes

In this section, we treat the fundamental solution $G_{\alpha ,\beta ,n}$ as a probability density function (with respect to the spatial variables) evolving in time (as mentioned above, it is the case for an arbitrary dimension n∈N if 0<β≤1,0<α≤2 and additionally for 1<β≤α≤2 in the one-dimensional case) and in particular derive the closed form formulas for the entropy and the entropy production rate of the underlying stochastic processes.

For an *n*-dimensional continuous random variable evolving in time with the probability density function p(x,t), $x\in R^{n}$, t>0, the Shannon entropy is defined by the formula

(26) $$S\left(p,t\right)=-\int_{R^{n}}p\left(x,t\right)\ln\left(p\left(x,t\right)\right)\;dx\;,$$

and the entropy production rate by its derivative

(27) $$R\left(p,t\right)=\frac{d}{dt}S\left(p,t\right)\;.$$

The entropy production rate is an important characteristic of a stochastic process that provides a measure of its irreversibility. For the conventional diffusion process (fundamental solution to the *n*-dimensional diffusion equation)

(28) $$G_{2,1,n}\left(x,t\right)=\frac{1}{{\left(\sqrt{4\pi t}\right)}^{n}}\exp\left(-\frac{{\left|x\right|}^{2}}{4t}\right)\;$$

the Shannon entropy is given by the formula

(29) $$S\left(G_{2,1,n},t\right)=\frac{n}{2}\left(\ln\left(4\pi t\right)+1\right).$$

The entropy production rate of the *n*-dimensional diffusion process is obtained by differentiation of the right-hand side of the Equation (29):

(30) $$R\left(G_{2,1,n},t\right)=\frac{d}{dt}S\left(G_{2,1,n},t\right)=\frac{n}{2t}.$$

The entropy production rate $R(G_{2,1,n},t)$ is strictly positive for any t>0 and thus the corresponding diffusion process can be classified as irreversible. Moreover, $R(G_{2,1,n},t)$ decreases with time and goes to zero for t→+∞ that can be interpreted in the sense that the diffusion process tends to a uniform distribution of the particles for t→+∞.

Now let us proceed with calculation of the Shannon entropy of the fractional diffusion processes governed by the multi-dimensional time-space-fractional diffusion Equation (1) under the restrictions on the orders of the fractional derivatives that ensure the non-negativity of its fundamental solution (see the previous section). To do this, we employ the representation (17) of its fundamental solution in terms of the auxiliary function $L_{\alpha ,\beta ,n}$.

Substituting (17) into (26) and using the well-known formula for the multi-dimensional integrals of the radial functions ([21])

(31) $$\int_{R^{n}}f\left(|x|\right)dx=\frac{2\pi^{\frac{n}{2}}}{\Gamma (\frac{n}{2})}\int_{0}^{\infty }\tau^{n-1}f\left(\tau \right)d\tau ,$$

we obtain the following chain of equalities:

$$\begin{array}{ccc} S(G_{\alpha ,\beta ,n},t) & = & -\int_{R^{n}}t^{-\frac{\beta n}{\alpha }}L_{\alpha ,\beta ,n}\left(\frac{x}{2t^{\beta /\alpha }}\right)\ln\left(t^{-\frac{\beta n}{\alpha }}L_{\alpha ,\beta ,n}\left(\frac{x}{2t^{\beta /\alpha }}\right)\right)\;dx\;\\ & = & -\frac{2\pi^{\frac{n}{2}}}{\Gamma \left(\frac{n}{2}\right)t^{\frac{\beta n}{\alpha }}}\int_{0}^{\infty }\tau^{n-1}L_{\alpha ,\beta ,n}\left(\frac{\tau }{2t^{\beta /\alpha }}\right)\left[-\frac{\beta n}{\alpha }\ln\left(t\right)+\ln\left(L_{\alpha ,\beta ,n}\left(\frac{\tau }{2t^{\beta /\alpha }}\right)\right)\right]\;d\tau \\ & = & \frac{2\pi^{\frac{n}{2}}}{\Gamma \left(\frac{n}{2}\right)t^{\frac{\beta n}{\alpha }}}\frac{\beta n}{\alpha }\ln\left(t\right)\int_{0}^{\infty }\tau^{n-1}L_{\alpha ,\beta ,n}\left(\frac{\tau }{2t^{\beta /\alpha }}\right)\;d\tau \\ & - & \frac{2\pi^{\frac{n}{2}}}{\Gamma \left(\frac{n}{2}\right)t^{\frac{\beta n}{\alpha }}}\int_{0}^{\infty }\tau^{n-1}L_{\alpha ,\beta ,n}\left(\frac{\tau }{2t^{\beta /\alpha }}\right)\;\ln\left(L_{\alpha ,\beta ,n}\left(\frac{\tau }{2t^{\beta /\alpha }}\right)\right)\;d\tau . \end{array}$$

In the last two integrals, we employ the variables substitution $\frac{\tau }{2t^{\beta /\alpha }}\to \tau$ and arrive at the formula

$$\begin{array}{cc} S(G_{\alpha ,\beta ,n},t) & =\frac{2^{n+1}\pi^{\frac{n}{2}}}{\Gamma \left(\frac{n}{2}\right)}\frac{\beta n}{\alpha }\ln\left(t\right)\int_{0}^{\infty }\tau^{n-1}L_{\alpha ,\beta ,n}\left(\tau \right)\;d\tau \\ & -\frac{2^{n+1}\pi^{\frac{n}{2}}}{\Gamma \left(\frac{n}{2}\right)}\int_{0}^{\infty }\tau^{n-1}L_{\alpha ,\beta ,n}\left(\tau \right)\;\ln\left(L_{\alpha ,\beta ,n}\left(\tau \right)\right)\;d\tau \end{array}$$

that can be represented in the form

(32) $$S\left(G_{\alpha ,\beta ,n},t\right)=A_{\alpha ,\beta ,n}\ln t+B_{\alpha ,\beta ,n}\;,$$

where the (time-independent) constants $A_{\alpha ,\beta ,n}$ and $B_{\alpha ,\beta ,n}$ are given by the expressions

(33) $$A_{\alpha ,\beta ,n}=\frac{2^{n+1}\pi^{\frac{n}{2}}}{\Gamma \left(\frac{n}{2}\right)}\frac{\beta n}{\alpha }\int_{0}^{\infty }\tau^{n-1}L_{\alpha ,\beta ,n}\left(\tau \right)\;d\tau ,$$

(34) $$B_{\alpha ,\beta ,n}=-\frac{2^{n+1}\pi^{\frac{n}{2}}}{\Gamma \left(\frac{n}{2}\right)}\int_{0}^{\infty }\tau^{n-1}L_{\alpha ,\beta ,n}\left(\tau \right)\;\ln\left(L_{\alpha ,\beta ,n}\left(\tau \right)\right)\;d\tau$$

in terms of the auxiliary function $L_{\alpha ,\beta ,n}$.

The representation (32) allows us to immediately calculate the entropy production rate of the *n*-dimensional fractional diffusion process governed by the time-space-fractional diffusion equation (1):

(35) $$R\left(G_{\alpha ,\beta ,n},t\right)=\frac{d}{dt}S\left(G_{\alpha ,\beta ,n},t\right)=\frac{A_{\alpha ,\beta ,n}}{t},$$

where the constant $A_{\alpha ,\beta ,n}$ is given by (33).

Surprisingly, the integral at the right-hand side of the Equation (33) can be calculated in explicit form. Indeed, this integral can be interpreted as the Mellin integral transform of the auxiliary function $L_{\alpha ,\beta ,n}$ at the point s=n. However, the Equation (25) for the Mellin integral transform of $L_{\alpha ,\beta ,n}$ was derived under the condition n−min(α,n)<Res<n and cannot be directly used at the point s=n. Thus we calculate the integral at the right-hand side of the Equation (33) as the limit of the right-hand side of the Equation (25) as s→n and get the following chain of equalities:

$$\begin{array}{ccc} \int_{0}^{\infty }\tau^{n-1}L_{\alpha ,\beta ,n}\left(\tau \right)\;d\tau & = & \lim_{s\to n}L_{\alpha ,\beta ,n}^{*}\left(s\right)\\ & = & \lim_{s\to n}\frac{1}{\alpha \;{\left(4\pi \right)}^{\frac{n}{2}}}\frac{\Gamma \left(\frac{s}{2}\right)\Gamma \left(\frac{n}{\alpha }-\frac{s}{\alpha }\right)\Gamma \left(1-\frac{n}{\alpha }+\frac{s}{\alpha }\right)}{\Gamma \left(1-\frac{\beta }{\alpha }n+\frac{\beta }{\alpha }s\right)\Gamma \left(\frac{n}{2}-\frac{s}{2}\right)}\\ & = & \lim_{s\to n}\frac{1}{\alpha \;{\left(4\pi \right)}^{\frac{n}{2}}}\frac{\Gamma \left(\frac{n}{2}\right)\Gamma \left(\frac{n-s}{\alpha }\right)\Gamma \left(1\right)}{\Gamma \left(1\right)\Gamma \left(\frac{n-s}{2}\right)}\\ & = & \lim_{s\to n}\frac{1}{\alpha \;{\left(4\pi \right)}^{\frac{n}{2}}}\frac{\Gamma \left(\frac{n}{2}\right)\frac{n-s}{2}}{\frac{n-s}{2\alpha }}\\ & = & \frac{\Gamma \left(\frac{n}{2}\right)}{2^{n+1}\pi^{\frac{n}{2}}}. \end{array}$$

The second to last equality is a simple consequence of the formula for the residual of the Gamma-function at the point s=0:

$${\mathrm{res}}_{s=0}\Gamma \left(s\right)=1.$$

Combining now the Equation (33) with the integral

$$\int_{0}^{\infty }\tau^{n-1}L_{\alpha ,\beta ,n}\left(\tau \right)\;d\tau =\frac{\Gamma \left(\frac{n}{2}\right)}{2^{n+1}\pi^{\frac{n}{2}}},$$

we arrive at the following formula for the constant $A_{\alpha ,\beta ,n}$:

(36) $$A_{\alpha ,\beta ,n}=\frac{2^{n+1}\pi^{\frac{n}{2}}}{\Gamma \left(\frac{n}{2}\right)}\frac{\beta n}{\alpha }\frac{\Gamma \left(\frac{n}{2}\right)}{2^{n+1}\pi^{\frac{n}{2}}}=\frac{\beta n}{\alpha }.$$

Thus the entropy production rate of the *n*-dimensional fractional diffusion process governed by the time-space-fractional diffusion Equation (1) can be represented in the following simple form:

(37) $$R\left(G_{\alpha ,\beta ,n},t\right)=\frac{\beta \;n}{\alpha \;t}.$$

As in the case of the conventional diffusion, the entropy production rate of the *n*-dimensional fractional diffusion process is non-negative and decreasing with time. Moreover, in the general case (for any dimension n∈N), the entropy production rate is an increasing function of the order of the time-fractional derivative β for β∈(0,1] as expected from the physical and probabilistic interpretations of the slow and conventional diffusion. Because β is restricted to the interval (0,1] (with the only exception for n=1), there exists no entropy production rate paradox.

Another important finding follows from comparison of the entropy production rates of the *n*-dimensional conventional diffusion (Equation (30)) and of the *n*-dimensional fractional diffusion process (Equation (37)). They are equal in the case

(38) $$\frac{\beta }{\alpha }=\frac{1}{2},$$

i.e., in the case of the α-fractional diffusion equation that was analyzed in detail in [16,17,18]. Thus the α-fractional diffusion equation can be treated as a “right fractionalization” of the conventional diffusion equation.

As one can see from the Equation (37), the entropy production rates and thus other properties of the fractional diffusion processes depend on the quotient $\frac{\beta }{\alpha }$ of the orders of the time- and space-fractional derivatives. Of course, it is a direct consequence of the form of the similarity variable in the representation (17) of the fundamental solution $G_{\alpha ,\beta ,n}$ in terms of the auxiliary function $L_{\alpha ,\beta ,n}$. From the other hand, the expression for the similarity variable is determined by the symmetries of the time-space-fractional PDE (1) with respect to the group of its scaling transformations (see [27,28] for discussion of the relevant particular cases and [29] for the general theory of the group invariants for the fractional PDEs).

## 4. Conclusions

In this paper, we derived and analyzed the fundamental solution to the *n*-dimensional time-space-fractional PDE with the Caputo time-fractional derivative of the order β,0<β<2 and the fractional spatial derivative (fractional Laplacian) of the order α,0<α≤2. This equation governs a fractional diffusion process if and only if its fundamental solution is non-negative and can be interpreted as a spatial pdf evolving in time that is the case for an arbitrary dimension n∈N if 0<β≤1,0<α≤2 and additionally for 1<β≤α≤2 if n=1. In all these cases, we derived an explicit formula for the entropy production rate of the fractional diffusion processes. It turned out that the rate depends on the orders β and α of the fractional time and spatial derivatives and on the dimension *n* and is given by the expression $\frac{\beta \;n}{\alpha \;t}$, *t* being the time variable.

The entropy production rate is an increasing function in β. However, one cannot speak about any entropy production paradoxes related to the fractional diffusion processes because the time-space-fractional PDE governs a fractional diffusion process in all dimensions only under the condition 0<β≤1, i.e., only in the cases of the slow and the conventional diffusion.

Another important finding is that the entropy production rate of the α-fractional diffusion equation (fractional PDE with the Caputo time-fractional derivative of order α,0<α≤1 and the fractional spatial derivative of order 2α) is equal to one of the conventional diffusion equations. Thus the α-fractional diffusion equation can be treated as a “right fractionalization” of the conventional diffusion equation.

It would be important to study the problems discussed in this paper for other types of the fractional PDEs. In particular, the fractional diffusion equations with the Riesz–Feller fractional spatial derivative and/or the Riemann–Liouville time-fractional derivative would be worth investigating.
